# Comprehensive analysis of metformin-associated lactic acidosis: Insights from the FDA Adverse Event Reporting System (FAERS)

**DOI:** 10.1097/MD.0000000000045007

**Published:** 2025-10-03

**Authors:** Jie Jin, Wukun Ge, Aiping Yu, Zhiyong Lan, Shuangli Zhang

**Affiliations:** a Department of Endocrinology, Ninghai First Hospital, Ninghai, China; b Department of Clinical Pharmacy, Ninghai First Hospital, Ninghai, China; c Department of Laboratory Medicine, The Third Hospital of Quzhou, Quzhou, China; d Department of Psychiatry, The Third Hospital of Quzhou, Quzhou, China; e Department of Science and Education, The Third Hospital of Quzhou, Quzhou, China.

**Keywords:** adverse drug events, drug interactions, FAERS, lactic acidosis, metformin, pharmacovigilance, risk factors

## Abstract

The present study aims to evaluate metformin-associated lactic acidosis (MALA) using real-world data from the Food and Drug Administration Adverse Event Reporting System (FAERS), providing reference for rational metformin use in clinical practice. Relevant adverse event data from January 2018 to March 2024 was extracted from the FAERS database. Disproportionality analysis, logistic regression, and time-to-event analysis were performed to assess risk factors, temporal patterns, and potential drug interactions associated with MALA. Among 40,883 metformin-related adverse event reports, 10,370 were MALA cases. Diabetes (adjusted odds ratio [OR]: 2.35, 95% confidence interval [CI]: 2.03–2.74), heart disease (adjusted OR: 1.75, 95% CI: 1.44–2.14), and hypertension (adjusted OR: 1.40, 95% CI: 1.21–1.62) were identified as significant risk factors. Contrast media showed the highest signal strength for drug interactions (reporting odds ratio = 323.7, 95% CI: 226.8–461.99), followed by furosemide and bisoprolol. The median time to lactic acidosis onset was 106 days, significantly longer than the 20 days for other adverse events (*P* < .0001), with 44.0% occurring within 30 days and 40.4% after 360 days of therapy initiation. Taken together, our comprehensive analysis of the FAERS database enhances understanding of MALA risk factors and temporal patterns, contributing to informed decision-making in metformin’s clinical application and facilitating timely management of this potentially life-threatening complication, particularly in patients with cardiovascular comorbidities.

## 1. Introduction

Metformin, a biguanide class medication, has been a cornerstone in the management of type 2 diabetes mellitus (T2DM) for decades. Its efficacy in glycemic control, coupled with its favorable effects on cardiovascular outcomes and weight management, has solidified its position as the first-line pharmacological therapy for T2DM.^[1,2]^ However, the use of metformin is not without risks, with lactic acidosis being one of the most serious, albeit rare, adverse events associated with its use.^[3,4]^

Metformin-associated lactic acidosis (MALA) is a potentially life-threatening condition characterized by elevated blood lactate levels (>5 mmol/L) and decreased blood pH (<7.35).^[5]^ While the incidence of MALA is reported to be low, ranging from 3 to 10 cases per 100,000 patient-years,^[6,7]^ its severity can manifest in dramatic clinical presentations including gastrointestinal symptoms, encephalopathy, hypotension, and rarely, transient complete blindness that resolves after hemodialysis treatment.^[8]^ These potentially fatal complications necessitate a thorough understanding of MALA’s risk factors and temporal patterns to facilitate early recognition and intervention. The pathophysiology of MALA is complex and not fully elucidated. It is postulated that metformin accumulation, particularly in patients with impaired renal function, can lead to inhibition of the mitochondrial respiratory chain complex I, resulting in increased anaerobic metabolism and lactate production.^[9,10]^ However, the interplay between metformin use and other potential risk factors in the development of lactic acidosis remains incompletely understood.

Previous studies have identified several risk factors for MALA, including renal impairment, hepatic dysfunction, and concomitant use of certain medications.^[11,12]^ However, these studies have often been limited by small sample sizes, retrospective designs, or focus on specific patient populations. Furthermore, the temporal relationship between metformin initiation and the onset of lactic acidosis has not been thoroughly investigated in large-scale studies. The Food and Drug Administration Adverse Event Reporting System (FAERS) provides a valuable resource for pharmacovigilance studies, offering a large-scale, real-world dataset of adverse drug events.^[13]^ While several studies have utilized FAERS data to investigate various drug safety concerns,^[14,15]^ a comprehensive analysis of MALA using this database has not been conducted to date.

Our study aims to address this gap by leveraging the FAERS database to conduct a comprehensive evaluation of the risk factors, temporal patterns, and potential drug interactions associated with MALA. By analyzing a large, diverse patient population, we seek to: identify and quantify risk factors for MALA, including comorbidities, concomitant medications, and demographic characteristics; investigate the temporal relationship between metformin initiation and the onset of lactic acidosis; explore potential drug–drug interactions that may increase the risk of MALA, with a particular focus on contrast agents and other commonly co-prescribed medications^[16]^; and compare the temporal patterns of MALA with other metformin-associated adverse events.

This comprehensive analysis will provide valuable insights into the risk profile of MALA, potentially informing clinical decision-making and guideline development for metformin use in high-risk populations. Moreover, by elucidating the temporal patterns of MALA onset, our findings may contribute to improved monitoring strategies and early intervention protocols for patients at elevated risk. In the following sections, we will detail our methodological approach to analyzing the FAERS data, present our findings on risk factors and temporal patterns of MALA, and discuss the implications of these results for clinical practice and future research directions.

## 2. Methods

### 2.1. Data source and study population

We conducted a retrospective pharmacovigilance study using data from the FAERS database between January 2018 and March 2024. FAERS contains adverse event reports submitted by healthcare professionals, consumers, and pharmaceutical manufacturers. The study included reports where metformin was listed as a primary suspect drug, excluding duplicates and reports with missing critical information such as event date or patient age. The primary outcome was MALA, identified using the Medical Dictionary for Regulatory Activities (MedDRA 27.1) preferred term “lactic acidosis.” Reports were classified into 2 groups based on the presence or absence of MALA.

### 2.2. Disproportionality analysis for drug interactions

To investigate the potential impact of concomitant medications on the risk of MALA, we conducted a disproportionality analysis using the reporting odds ratio (ROR) and information component (IC). ROR compares the odds of MALA reporting for a specific drug–drug interaction with the odds of MALA reporting for all other drug pairs. IC is a Bayesian measure that compares the observed frequency of a drug–event combination to the expected frequency under the assumption of independence. Positive ROR and IC values suggest a higher-than-expected reporting rate for a given drug–event pair. We focused on concomitant medications commonly used in patients with type 2 diabetes and those previously associated with lactic acidosis.^[17,18]^

### 2.3. Variables and statistical analysis

The analysis included measures such as median (IQR) for continuous variables and frequencies and percentages for categorical variables. Group comparisons for continuous variables were performed using the Wilcoxon rank-sum test or the Kruskal–Wallis test. For comparison between groups of categorical data, we used the Fisher exact test for expected frequencies < 5; otherwise, we used the Chi-squared test.

Univariate logistic regression analysis was performed to assess the association between each individual factor (sex, age, weight, diabetes, hypertension, kidney, heart disease, lipids, depression, asthma, indi_frequency, and drug_frequency) and outcome variable result. This step helped identify factors that exhibited a potential relationship with the outcome variable. Multivariate logistic regression analysis was performed to determine the independent factors significantly associated with the result, while adjusting for potential confounders. A backward stepwise regression method, specifically the stepAIC function from the MASS package in R (version 4.2.2; R Foundation for Statistical Computing, Vienna, Austria), was employed to select the best combination of variables from the univariate analysis for inclusion in the multivariate analysis model. The selection process was based on minimizing the AIC value, which measures the goodness-of-fit and complexity of the model. The backward stepwise regression procedure involved starting with a model that included all potential variables, and then iteratively removing variables to find the optimal combination that minimized the AIC value. This method provided insights into the complex relationships among variables while optimizing model selection based on the AIC criterion using a backward elimination approach. In our study, all statistical analyses were performed using the R software (version 4.2.2), along with MSTATA software (www.mstata.com).

### 2.4. Time-to-event analysis

**Figure 1.**
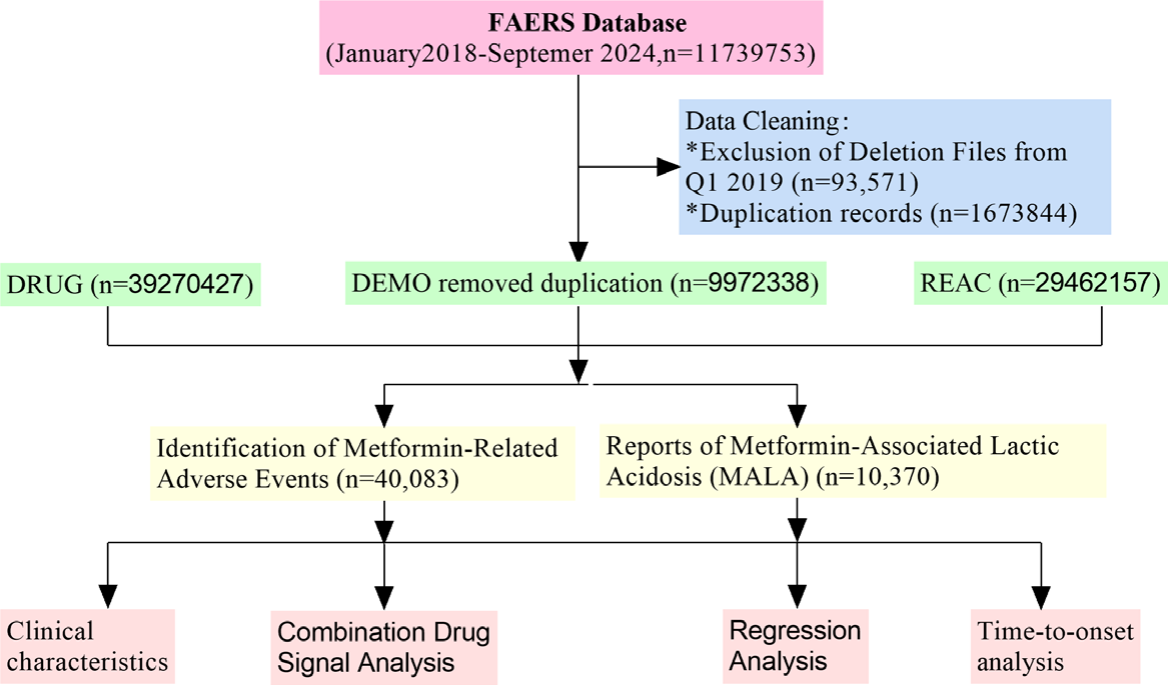
Flowchart of the metformin-associated lactic acidosis (MALA) study using the FDA Adverse Event Reporting System (FAERS) database. DEMO = demographic data; MALA = metformin-associated lactic acidosis; REAC = adverse reaction records.

The Weibull accelerated failure time model was used to compare the distribution of time from metformin initiation to the occurrence of MALA versus other adverse events. Survival curves were plotted, and differences were assessed using the likelihood ratio test. We acknowledge the limitations of this approach, particularly the absence of participants where no events occurred, which precludes the estimation of true incidence rates. However, the Weibull model was chosen for its flexibility in modeling time-to-event data in pharmacovigilance studies, where the focus is on relative risks rather than absolute incidence.^[17]^ Alternative methods, such as Poisson regression, were considered but deemed less suitable given the nature of the data and the research questions addressed. Figure 1 illustrates the overall study design and data analysis process, including the identification of metformin-associated adverse event reports, classification of MALA cases, descriptive and inferential statistical analyses, time-to-event analysis, and disproportionality analysis for drug interactions. The analysis results of baseline plots and temporal analysis plots were generated using the CNSknowall platform (https://cnsknowall.com), a comprehensive web service for data analysis and visualization.

### 2.5. Ethical considerations

This study used de-identified data from the publicly available FAERS database and was exempt from Institutional Review Board approval.

## 3. Result

### 3.1. Baseline characteristics of adverse event reports

**Figure 2.**
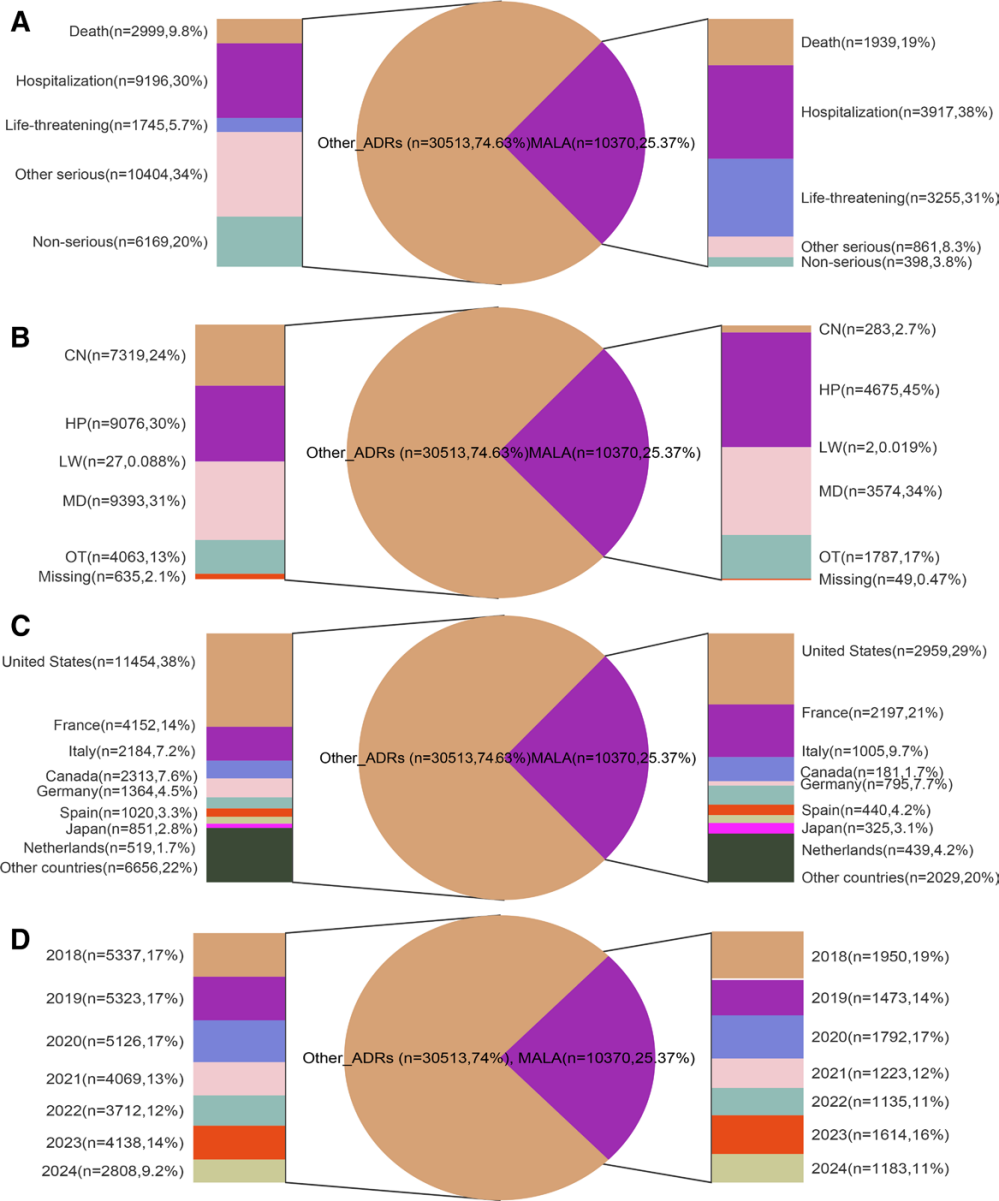
Illustrates the characteristics of metformin-associated adverse drug reactions reported in the FAERS database. The 4 sub-figures represent seriousness distribution (A), reporting sources (B) with CN (consumers), HP (Pharmacist), LW (lawyers), MD (physicians), and OT (Other health-professional), geographic distribution (C), and annual reporting trends from 2018 to 2024 (D).

Based on the data presented in Table 1 and Figure 2, this study analyzed a large real-world dataset of 40,883 adverse drug reaction (ADR) reports related to metformin, revealing key epidemiological characteristics of MALA compared to other ADRs. The results highlight that advanced age (65–85 years) is a significant risk factor for MALA (*P* < .001), with MALA cases having a notably higher proportion of elderly patients compared to other ADRs (44.2% vs 31.8%). Regarding weight, although the overall weight distribution showed a significant difference between groups (*P* < .001), specific patterns were less clear with substantial missing data (76.7%). Both lower weight (<50 kg) and higher weight (>100 kg) categories showed slightly lower proportions in MALA cases compared to other ADRs (0.9% vs 1.6% and 4.4% vs 4.9%, respectively). Furthermore, Figure 2 unveils important patterns: MALA leads to a markedly higher percentage of deaths and life-threatening outcomes; hospital pharmacists (HP) report nearly half of MALA cases, while physicians (MD) report the most for other ADRs; although the United States is the primary reporting country overall, France has a higher reporting proportion for MALA compared to other ADRs; and ADR and MALA reports have declined in recent years, particularly during 2021 to 2022, possibly due to the impact of the COVID-19 pandemic. These findings underscore the crucial role of healthcare institutions and pharmacists in monitoring MALA, and the evolving trends in ADR reporting during the pandemic, prompting further research directions and improvement strategies in areas such as clinical medication monitoring, pharmaceutical care, and pharmacovigilance.

**Table 1:** Baseline characteristics of metformin adverse event and lactic acidosis reports.

Characteristic	Total ADRs	Other ADRs	MALA	*P*-value
Gender	N = 40883	N = 30513	N = 10,370	*P*>.05
Female	19,310 (47.2%)	14,658 (48.0%)	4652(44.9%)	
Male	15,805 (38.7%)	11,867 (38.9%)	3938(38.0%)	
Missing	5768 (14.1%)	3988 (13.1%)	1780(17.2%)	
Weight				*P*<.001
<50 kg	577 (1.4%)	479 (1.6%)	98 (0.9%)	
>100 kg	1944 (4.8%)	1488 (4.9%)	456 (4.4%)	
50–100 kg	7578 (18.5%)	5720 (18.7%)	1858 (17.9%)	
Missing	30,784 (75.3%)	22,826 (74.8%)	7958 (76.7%)	
Age (yr)				*P*<.001
<18	660 (1.6%)	486 (1.6%)	174 (1.7%)	
>85	1306 (3.2%)	938 (3.1%)	368 (3.5%)	
18–64.9	13,271 (32.5%)	9922 (32.5%)	3349 (32.3%)	
65–85	14,301 (35.0%)	9718 (31.8%)	4583 (44.2%)	
Missing	11,345 (27.8%)	9449 (31.0%)	1896 (18.3%)	

Other ADRs: adverse drug reactions excluding MALA (metformin-associated lactic acidosis).

MALA = metformin-associated lactic acidosis.

*P*-values calculated using *χ*² test; significance level α = 0.05.

### 3.2. Disproportionality analysis for drug interactions

The signal detection analysis of FAERS data (Table S1, Supplemental Digital Content, https://links.lww.com/MD/Q213) revealed that metformin had the highest number of reported lactic acidosis cases (a = 10,374) and the strongest signal (ROR = 353.96, 95% confidence interval [CI]: 342.92–365.36; proportional reporting ratio = 328.81, χ² = 1287,144.9; empirical Bayesian geometric mean [EBGM] = 125.38, EBGM05 = 122.1; IC = 6.97, IC025 = 6.93), as calculated using the formulas and signal detection criteria shown in Table S2, Supplemental Digital Content, https://links.lww.com/MD/Q213. Among the concomitant medications, contrast media showed the highest signal strength (ROR = 323.7, 95% CI: 226.8–461.99; proportional reporting ratio = 273.62, χ² = 9763.12; EBGM = 273.04, EBGM05 = 202.75; IC = 8.09, IC025 = 7.58), followed by furosemide, bisoprolol, and amlodipine (Table 2), suggesting an increased risk of lactic acidosis when these medications are used in combination with metformin.

**Table 2:** Signal detection results of lactic acidosis caused by metformin and concomitant medications in the FAERS database.

Drug report	Case	ROR (95% Cl)	PRR (χ²)	EBGM (EBGM05)	IC (IC025)
Metformin	10,370	353.96 (342.92–365.36)	328.81 (1287144.9)	125.38 (122.1)	6.97 (6.93)
Contrast media	36	323.7 (226.8–461.99)	273.62 (9763.12)	273.04 (202.75)	8.09 (7.58)
Aspirin	820	103.06 (95.9–110.75)	97.67 (74649.46)	92.93 (87.49)	6.54 (6.43)
Bisoprolol	702	125.15 (115.76–135.31)	117.22 (77540.8)	112.34 (105.25)	6.81 (6.7)
Allopurinol	240	88.15 (77.36–100.44)	84.04 (19421.86)	82.85 (74.28)	6.37 (6.18)
Gliclazide	449	75.38 (68.5–82.95)	72.4 (30784.69)	70.48 (65.06)	6.14 (6)
Pantoprazole	425	78 (70.69–86.06)	74.81 (30178.57)	72.93 (67.17)	6.19 (6.04)
Sitagliptin	582	105.51 (96.91–114.87)	99.79 (54967.15)	96.35 (89.73)	6.59 (6.47)
Insulin glargine	390	70.5 (63.64–78.09)	67.88 (25112.1)	66.32 (60.87)	6.05 (5.9)
Atorvastatin	928	113.97 (106.47–122)	107.45 (92486.64)	101.54 (95.92)	6.67 (6.57)
Amlodipine	719	115.37 (106.82–124.59)	108.61 (73403.24)	103.98 (97.5)	6.7 (6.59)
Furosemide	789	155.4 (144.27–167.39)	143.41 (106369.11)	136.68 (128.45)	7.09 (6.99)

CI = confidence interval, EBGM = empirical Bayesian geometric mean, EBGM05 = the lower limit of 95% CI of EBGM, IC = information component, IC025 = the lower limit of 95% CI of the IC, PRR = proportional reporting ratio, ROR = reporting odds ratio, χ^2^ = chi-square.

### 3.3. Variables and statistical analysis

**Figure 3.**
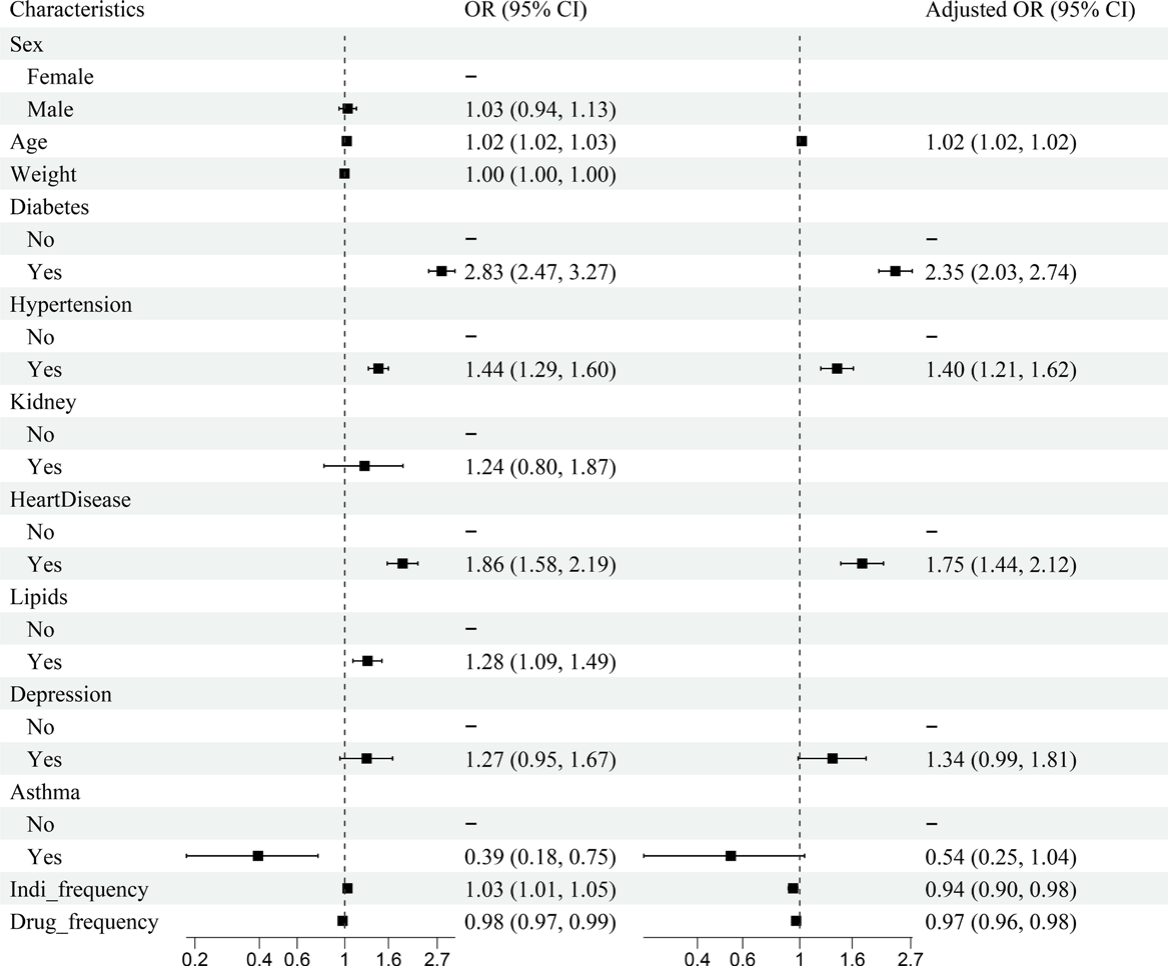
Forest plot of odds ratios (ORs) for factors associated with metformin-induced lactic acidosis (MALA). The plot shows unadjusted (OR) and adjusted odds ratios (adjusted OR) with 95% confidence intervals (CI) for various patient characteristics and comorbidities. The reference category for each characteristic is indicated by a dash (-). ORs to the left of the vertical line at 1.0 indicate a lower risk, while those to the right indicate a higher risk of lactic acidosis. Indi_frequency represents the frequency of indications, and drug_frequency represents the frequency of concomitant drugs. Adjustments were made for potential confounding factors in the multivariate analysis.

Based on the results presented in Table 3 and Figure 3, several significant associations emerged between patient characteristics and outcomes. Among the 9212 patients analyzed (2358 cases, 6854 controls), diabetes demonstrated the strongest association (adjusted odds ratio [OR]: 2.35, 95% CI: 2.03–2.74). This was followed by heart disease (adjusted OR: 1.75, 95% CI: 1.44–2.12; 10.6% vs 6.0%, *P* < .001) and hypertension (adjusted OR: 1.40, 95% CI: 1.21–1.62; 27.6% vs 21.0%, *P* < .001). Age showed a modest positive association (adjusted OR: 1.02, 95% CI: 1.02–1.02), with cases being significantly older than controls (68 ± 12 vs 63 ± 16 years, *P* < .001). Interestingly, asthma exhibited an inverse relationship (adjusted OR: 0.54, 95% CI: 0.25–1.04), and both indi_frequency (adjusted OR: 0.94, 95% CI: 0.90–0.98) and drug_frequency (adjusted OR: 0.97, 95% CI: 0.96–0.98) demonstrated slight negative associations. The multicollinearity diagnostics confirmed model robustness, with all variables showing variance inflation factors below 3.1 and tolerance values above 0.3, indicating minimal collinearity issues (Table S3, Supplemental Digital Content, https://links.lww.com/MD/Q213).

**Table 3:** Patient demographics and baseline characteristics.

Characteristic	Result		*P*-value
	Yes, N = 2,358^1^	No, N = 6,854*	
Sex			.499†
Female	1172 (49.7%)	3462 (50.5%)	
Male	1186 (50.3%)	3392 (49.5%)	
Age	68 ± 12	63 ± 16	<.001‡
Weight	83 ± 23	85 ± 26	.006‡
Diabetes			<.001†
Yes	2102 (89.1%)	5096 (74.4%)	
No	256 (10.9%)	1758 (25.6%)	
Hypertension			<.001†
No	1708 (72.4%)	5418 (79.0%)	
Yes	650 (27.6%)	1436 (21.0%)	
Kidney			.322†
No	2327 (98.7%)	6781 (98.9%)	
Yes	31 (1.3%)	73 (1.1%)	
Heart disease			<.001†
No	2107 (89.4%)	6442 (94.0%)	
Yes	251 (10.6%)	412 (6.0%)	
Lipids			.002†
No	2106 (89.3%)	6267 (91.4%)	
Yes	252 (10.7%)	587 (8.6%)	
Depression			.100†
No	2287 (97.0%)	6690 (97.6%)	
Yes	71 (3.0%)	164 (2.4%)	
Asthma			.007†
No	2349 (99.6%)	6788 (99.0%)	
Yes	9 (0.4%)	66 (1.0%)	
Indi_frequency	2.55 ± 2.05	2.43 ± 2.02	.010‡
Drug_frequency	5.6 ± 4.6	6.1 ± 5.5	<.001‡

* n (%), mean ± SD.

† Pearson Chi-squared test.

‡ Welch 2-sample *t* test.

### 3.4. Time-to-event analysis

**Figure 4.**
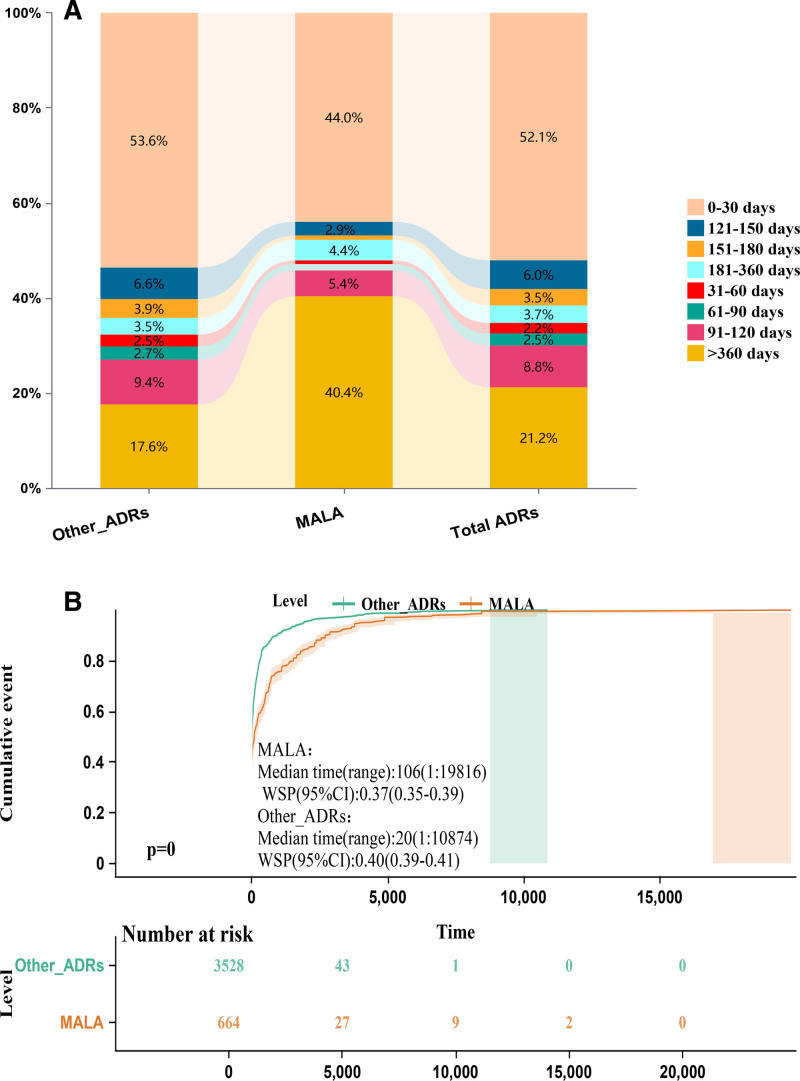
Illustrates the time-to-onset distribution and survival analysis of metformin-associated lactic acidosis (MALA) and other adverse drug reactions (ADRs) reported in the FAERS database, using a categorized time-to-onset graph (A) and a Kaplan–Meier plot (B) with the median time-to-onset and cumulative incidence at the median time (WSP) for both MALA and other ADRs.

Figure 4 presents a time-to-event analysis of metformin-associated ADRs using FAERS data, revealing distinct temporal patterns between lactic acidosis (MALA, n = 664) and other ADRs (n = 3528). Panel A shows 44.0% of MALA cases reported within 30 days of metformin initiation and 40.4% after 360 days, highlighting the need for vigilant monitoring throughout therapy. Panel B’s Kaplan–Meier curves demonstrate a significantly longer median time to onset for MALA (106 days, range: 1–19,816) compared to other ADRs (20 days, range: 1–10,874), with early curve divergence sustained during follow-up (log-rank test, *P* < .0001). These findings underscore the importance of promptly recognizing and managing MALA, particularly given its potential for delayed onset and long-term risk, to inform clinical decision-making and risk mitigation strategies for metformin-treated patients.

## 4. Discussion

This comprehensive analysis of MALA using the FAERS database provides critical insights into the risk factors, temporal patterns, and potential drug interactions associated with this rare but serious adverse event. Our findings have important implications for clinical practice and future research directions managing patients on metformin therapy.

Our study identified several significant risk factors for MALA, including diabetes, heart disease, and hypertension, which show the strongest associations. The high odds ratio for diabetes (adjusted OR: 2.35, 95% CI: 2.03–2.74) is not unexpected, given that metformin is primarily prescribed for T2DM. However, this finding underscores the importance of vigilant monitoring for MALA in diabetic patients, particularly those with long-standing or poorly controlled disease. The association between diabetes and MALA may be explained by the increased risk of renal impairment in diabetic patients, which can lead to metformin accumulation and subsequent lactic acidosis.^[12,19]^ The strong associations between cardiovascular comorbidities and MALA align with previous studies.^[20,21]^ Heart disease (adjusted OR: 1.75, 95% CI: 1.44–2.12) may increase MALA risk due to reduced tissue perfusion and oxygen delivery, exacerbating metformin’s effects on lactate production.^[22]^ Hypertension (adjusted OR: 1.40, 95% CI: 1.21–1.62) often coexists with diabetes and cardiovascular disease, and is a risk factor for chronic kidney disease, potentially impairing metformin clearance.^[6]^ Interestingly, indication frequency (adjusted OR: 0.94, 95% CI: 0.90–0.98) and drug frequency (adjusted OR: 0.97, 95% CI: 0.96–0.98) showed inverse associations with MALA risk. This counterintuitive finding may be due to our analysis being confined to patients who reported adverse events with metformin. Possible explanations include selection bias, where frequent users are under closer medical supervision, and survivor bias, where those tolerating frequent use may be less susceptible to MALA.^[23]^ These results highlight the complex interplay between metformin use, comorbidities, and MALA risk. Recent studies have shown the potential cardiovascular benefits of metformin in specific populations,^[24,25]^ emphasizing the need for personalized risk assessment and management strategies in metformin therapy, particularly for patients with cardiovascular comorbidities.^[26]^

Our time-to-event analysis revealed important temporal patterns in the onset of MALA compared to other metformin-associated adverse events. The median time to lactic acidosis onset was significantly longer (106 days) than for other adverse events (20 days), with a wide range extending up to 19,816 days. This finding has several important implications: early-decay pattern: both MALA and other adverse events showed higher occurrence risks in the initial stages of treatment. This underscores the importance of close monitoring during the 1st few months of metformin therapy, particularly in patients with identified risk factors. Delayed onset of MALA: the significantly longer median time to MALA onset suggests that the risk of lactic acidosis persists well beyond the initial treatment period. This finding challenges the common clinical practice of focusing primarily on early adverse events and highlights the need for ongoing vigilance throughout the course of metformin therapy. Long-term risk: the extended range of MALA onset times (up to 19,816 days) indicates that some patients may develop lactic acidosis even after years of apparently safe metformin use. This emphasizes the importance of regular reassessment of MALA risk factors in long-term metformin users, particularly as they age and potentially develop new comorbidities. These temporal patterns align with the findings of Lalau et al,^[3]^ who proposed a new paradigm for understanding MALA that emphasizes the role of acute precipitating events in long-term metformin users. Our results support this concept and suggest that clinicians should maintain a high index of suspicion for MALA throughout the duration of metformin therapy, particularly in the setting of acute illness or changes in renal function.

Our disproportionality analysis revealed several important potential drug interactions that may increase the risk of MALA. The strongest signal was observed for contrast media (ROR = 323.7, 95% CI: 226.8–461.99), which aligns with long-standing concerns about the use of iodinated contrast in patients taking metformin.^[27,28]^ This finding reinforces the importance of current guidelines recommending temporary discontinuation of metformin around the time of contrast administration, particularly in patients with renal impairment.^[29]^ Other medications showing strong signals for potential interaction with metformin included furosemide, bisoprolol, and amlodipine. The association with furosemide (ROR = 155.4, 95% CI: 144.27–167.39) is particularly noteworthy, as loop diuretics can affect renal function and electrolyte balance, potentially exacerbating the risk of lactic acidosis.^[30]^ The signals for bisoprolol and amlodipine may reflect the underlying cardiovascular comorbidities in patients receiving these medications rather than direct pharmacological interactions. However, the possibility of additive effects on cellular metabolism and lactate production cannot be ruled out and warrants further investigation.

These findings highlight the complex interplay between metformin, comorbidities, and concomitant medications in the pathogenesis of MALA. Clinicians should be aware of these potential interactions and exercise caution when prescribing metformin in combination with these medications, particularly in patients with other risk factors for lactic acidosis.

While our study provides valuable insights into the risk factors and patterns of MALA, several limitations should be acknowledged. First, the FAERS database relies on spontaneous reporting, which can be subject to reporting biases and underestimation of true adverse event rates.^[31]^ Second, the database lacks detailed information on patient characteristics, medication dosages, and laboratory values, which limits our ability to fully adjust for potential confounders and assess dose–response relationships. Third, our study identifies MALA cases based on adverse event outcomes reported to FAERS rather than standardized clinical and laboratory diagnostic criteria, potentially introducing diagnostic accuracy limitations. This diagnostic concern is further reflected in the reporting source distribution shown in Figure 2B, where hospital pharmacists (HP) contribute a notably higher proportion of MALA reports (45%) compared to other ADRs (30%), while physician (MD) reporting increases less dramatically (from 31–34%), suggesting variations in diagnostic approaches and reporting practices across healthcare professionals. Future research should focus on addressing these limitations through prospective studies and the integration of multiple data sources. Large-scale, longitudinal cohort studies that combine electronic health records, claims data and pharmacovigilance databases could provide more comprehensive insights into the long-term risks and predictors of MALA.^[32,33]^ Additionally, pharmacogenomic studies may help identify genetic factors that predispose certain individuals to MALA, potentially leading to more personalized risk assessment and management strategies.^[34]^ Given the observed temporal patterns of MALA onset, future studies should also investigate the role of acute precipitating factors in long-term metformin users. This could involve detailed case–control studies examining the clinical circumstances surrounding MALA events in patients with extended metformin use.

In conclusion, our comprehensive analysis of MALA using the FAERS database has revealed important risk factors, temporal patterns, and potential drug interactions associated with this serious adverse event. These findings underscore the need for ongoing vigilance throughout the course of metformin therapy, particularly in patients with multiple comorbidities and concomitant medications. By integrating these insights into clinical practice and future research efforts, we can work towards optimizing the safe and effective use of metformin in the management of type 2 diabetes mellitus.

## Author contributions

**Conceptualization:** Jie Jin.

**Data curation:** Wukun Ge, Aiping Yu, Zhiyong Lan.

**Formal analysis:** Zhiyong Lan.

**Methodology:** Zhiyong Lan.

**Resources:** Wukun Ge.

**Supervision:** Shuangli Zhang.

**Writing – original draft:** Jie Jin, Shuangli Zhang.

**Writing – review & editing:** Shuangli Zhang.

## Supplementary Material

## References

[1] Yao L Wang L Zhang R Soukas AA Wu L. The direct targets of metformin in diabetes and beyond. Trends Endocrinol Metab. 2025;36:364–72. 39227192 10.1016/j.tem.2024.07.017 PMC12585531

[2] Stone C Sabe SA Harris DD. Metformin preconditioning augments cardiac perfusion and performance in a large animal model of chronic coronary artery disease. Ann Surg. 2024;280:547–56. 39041226 10.1097/SLA.0000000000006437

[3] Lalau J-D Kajbaf F Protti A Christensen MM De Broe ME Wiernsperger N. Metformin-associated lactic acidosis (MALA): Moving towards a new paradigm. Diabetes Obes Metab. 2017;19:1502–12. 28417525 10.1111/dom.12974

[4] DeFronzo R Fleming GA Chen K Bicsak TA. Metformin-associated lactic acidosis: Current perspectives on causes and risk. Metabolism. 2016;65:20–9. 26773926 10.1016/j.metabol.2015.10.014

[5] Bakris GL Molitch ME. Should restrictions be relaxed for metformin use in chronic kidney disease? Yes, they should be relaxed! What’s the fuss? Diabetes Care. 2016;39:1287–91. 27330130 10.2337/dc15-2534

[6] Salpeter SR Greyber E Pasternak GA Salpeter EE. Risk of fatal and nonfatal lactic acidosis with metformin use in type 2 diabetes mellitus. Cochrane Database Syst Rev. 2010;2010:CD002967. 20091535 10.1002/14651858.CD002967.pub3

[7] Inzucchi SE Lipska KJ Mayo H Bailey CJ McGuire DK. Metformin in patients with type 2 diabetes and kidney disease: a systematic review. JAMA. 2014;312:2668–75. 25536258 10.1001/jama.2014.15298 PMC4427053

[8] Prada LR Knopps L Dumic I. Transient complete blindness due to Metformin-Associated Lactic Acidosis (MALA) reversed with hemodialysis. Am J Case Rep. 2022;23:e935730. 35431313 10.12659/AJCR.935730 PMC9026230

[9] Bridges HR Jones AJY Pollak MN Hirst J. Effects of metformin and other biguanides on oxidative phosphorylation in mitochondria. Biochem J. 2014;462:475–87. 25017630 10.1042/BJ20140620 PMC4148174

[10] Andrzejewski S Gravel S-P Pollak M St-Pierre J. Metformin directly acts on mitochondria to alter cellular bioenergetics. Cancer Metab. 2014;2:12. 25184038 10.1186/2049-3002-2-12 PMC4147388

[11] Cucchiari D Podestà MA Merizzoli E. Dose-related effects of metformin on acid-base balance and renal function in patients with diabetes who develop acute renal failure: a cross-sectional study. Acta Diabetol. 2016;53:551–8. 26821225 10.1007/s00592-016-0836-2

[12] Hung S-C Chang Y-K Liu J-S. Metformin use and mortality in patients with advanced chronic kidney disease: national, retrospective, observational, cohort study. Lancet Diabetes Endocrinol. 2015;3:605–14. 26094107 10.1016/S2213-8587(15)00123-0

[13] Zhou J Zheng Y Xu B. Exploration of the potential association between GLP-1 receptor agonists and suicidal or self-injurious behaviors: a pharmacovigilance study based on the FDA adverse event reporting system database. BMC Med. 2024;22:65. 38355513 10.1186/s12916-024-03274-6 PMC10865629

[14] Salah S Kerob D Pages Laurent C Lacouture M Sibaud V. Evaluation of anticancer therapy-related dermatologic adverse events: insights from food and drug administration’s adverse event reporting system dataset. J Am Acad Dermatol. 2024;91:863–71. 39038557 10.1016/j.jaad.2024.07.1456

[15] Ge W Chen W Xie H. Real-world evidence supporting the consensus-driven DILI control compounds list: an analysis of FAERS data. J Hepatol. 2025;82:e204–5. 39461595 10.1016/j.jhep.2024.10.030

[16] Thomsen HS Morcos SK. Contrast media and metformin: guidelines to diminish the risk of lactic acidosis in non-insulin-dependent diabetics after administration of contrast media. ESUR contrast media safety committee. Eur Radiol. 1999;9:738–40. 10354898 10.1007/s003300050746

[17] Salem J-E Manouchehri A Moey M. Cardiovascular toxicities associated with immune checkpoint inhibitors: an observational, retrospective, pharmacovigilance study. Lancet Oncol. 2018;19:1579–89. 30442497 10.1016/S1470-2045(18)30608-9 PMC6287923

[18] Salem J-E Manouchehri A Bretagne M. Cardiovascular toxicities associated with ibrutinib. J Am Coll Cardiol. 2019;74:1667–78. 31558250 10.1016/j.jacc.2019.07.056

[19] Yi Y Kwon E-J Yun G. Impact of metformin on cardiovascular and kidney outcome based on kidney function status in type 2 diabetic patients: a multicentric, retrospective cohort study. Sci Rep. 2024;14:2081. 38267451 10.1038/s41598-024-52078-4 PMC10808543

[20] Zhang L Zhao X Wang Z. Preadmission metformin use increased the incidence of hyperlactatemia at admission and 30-day in-hospital mortality among T2D patients with heart disease at high risk of hypoxia. Int J Cardiol. 2024;412:132338. 38964551 10.1016/j.ijcard.2024.132338

[21] Salvatore T Pafundi PC Marfella R. Metformin lactic acidosis: should we still be afraid? Diabetes Res Clin Pract. 2019;157:107879. 31618624 10.1016/j.diabres.2019.107879

[22] Salvatore T Pafundi PC Galiero R. Metformin: a potential therapeutic tool for rheumatologists. Pharmaceuticals (Basel). 2020;13:234. 32899806 10.3390/ph13090234 PMC7560003

[23] Suissa S Azoulay L. Metformin and cancer: mounting evidence against an association. Diabetes Care. 2014;37:1786–8. 24963109 10.2337/dc14-0500

[24] Campbell JM Stephenson MD de Courten B Chapman I Bellman SM Aromataris E. Metformin use associated with reduced risk of dementia in patients with diabetes: a systematic review and meta-analysis. J Alzheimers Dis. 2018;65:1225–36. 30149446 10.3233/JAD-180263 PMC6218120

[25] Lamanna C Monami M Marchionni N Mannucci E. Effect of metformin on cardiovascular events and mortality: a meta-analysis of randomized clinical trials. Diabetes Obes Metab. 2011;13:221–8. 21205121 10.1111/j.1463-1326.2010.01349.x

[26] Li S Vandvik PO Lytvyn L. SGLT-2 inhibitors or GLP-1 receptor agonists for adults with type 2 diabetes: a clinical practice guideline. BMJ. 2021;373:n1091. 33975892 10.1136/bmj.n1091

[27] Sakellariou XM Kolettis TM Nikas DN. Renal complications after percutaneous coronary interventions on concurrent metformin therapy: a systematic review with meta-analysis. Clin Med Res. 2023;21:26–35. 37130786 10.3121/cmr.2022.1759 PMC10153679

[28] Goergen SK Rumbold G Compton G Harris C. Systematic review of current guidelines, and their evidence base, on risk of lactic acidosis after administration of contrast medium for patients receiving metformin. Radiology. 2010;254:261–9. 20032157 10.1148/radiol.09090690

[29] Kløw NE Draganov B Os I. Metformin and contrast media – increased risk of lactic acidosis?. Tidsskr Nor Laegeforen. 2001;121:1829. 11464691

[30] Rojas LBA Gomes MB. Metformin: an old but still the best treatment for type 2 diabetes. Diabetol Metab Syndr. 2013;5:6. 23415113 10.1186/1758-5996-5-6 PMC3607889

[31] Sarangdhar M Tabar S Schmidt C. Data mining differential clinical outcomes associated with drug regimens using adverse event reporting data. Nat Biotechnol. 2016;34:697–700. 27404875 10.1038/nbt.3623 PMC12911468

[32] Litvinova O Yeung AWK Hammerle FP. Digital technology applications in the management of adverse drug reactions: bibliometric analysis. Pharmaceuticals (Basel). 2024;17:395. 38543181 10.3390/ph17030395 PMC10975491

[33] Mao C Yao L MedGCN LY. Medication recommendation and lab test imputation via graph convolutional networks. J Biomed Inform. 2022;127:104000. 35104644 10.1016/j.jbi.2022.104000 PMC8901567

[34] Dujic T Causevic A Bego T. Organic cation transporter 1 variants and gastrointestinal side effects of metformin in patients with Type 2 diabetes. Diabet Med. 2016;33:511–4. 26605869 10.1111/dme.13040 PMC5064645

