# Supplementary material for: Comprehensive analysis of metformin-associated lactic acidosis: Insights from the FDA Adverse Event Reporting System (FAERS)
Source: Medicine (Baltimore). 2025 Oct 3;104(40):e45007. doi: 10.1097/MD.0000000000045007 (PMC12499714; doi:10.1097/MD.0000000000045007)
Supplement: Supplementary file 1 [file medi-104-e45007-s001.docx]

**Supplementary table 1**. Contingency table of adverse reactions with metformin and concomitant drugs.

| Drugs | Target adverse event | Other adverse events | Total |
| --- | --- | --- | --- |
| Patients on metformin PS with concomitant Drug B (SS, I, C) | a | b | a+b |
| Total population minus patients on metformin PS with concomitant Drug B (SS, I, C) | c | d | c+d |
| Total | a+c | b+d | N=a+b+c+d |

**Supplementary table 2** Disproportionality analysis methods, formulas, and signal detection criteria

| Disproportionality Methods | Equation | Signal Detection Criteria |
| --- | --- | --- |
| Reporting Odds Ratio (ROR) | ROR=ad/b/c | lower limit of 95% CI>1, N≥3 |
|  | 95%CI=e^ln(ROR)±1.96(1/a+1/b+1/c+1/d)^0.5^ |  |
| Proportional Reporting Ratio(PRR) | PRR=a(c+d)/c/(a+b) | PRR≥2, χ^2^≥4, N≥3 |
|  | χ^2^=[(ad-bc)^2](a+b+c+d)/[(a+b)(c+d)(a+c)(b+d)] |  |
| Bayesian Confidence Propagation Neural Network | IC=log_2_a(a+b+c+d)(a+c)(a+b) | IC025>0 |
|  | 95%CI= E(IC) ± 2V(IC)^0.5 |  |
| Empirical Bayesian Geometric Mean | EBGM=a(a+b+c+d)/(a+c)/(a+b) | EBGM05>2 |
|  | 95%CI=e^ln(EBGM)±1.96(1/a+1/b+1/c+1/d)^0.5^ |  |

CI: Confidence interval; N: Number of reports; χ²: Chi-squared; IC: Information component; IC025: Lower limit of 95% CI of the IC; E(IC): Expected value of IC; V(IC): Variance of IC; EBGM: Empirical Bayesian geometric mean; EBGM05: Lower limit of 95% CI of EBGM.

**Supplementary table 3** :Variance Inflation Factor and Tolerance

| Term | VIF | VIF_CI_low | VIF_CI_high | SE_factor | Tolerance | Tolerance_CI_low | Tolerance_CI_high |
| --- | --- | --- | --- | --- | --- | --- | --- |
| Age | 1.052567 | 1.034237 | 1.080710 | 1.025947 | 0.9500583 | 0.9253175 | 0.9668962 |
| Diabetes | 1.110787 | 1.088475 | 1.138725 | 1.053939 | 0.9002626 | 0.8781751 | 0.9187162 |
| Hypertension | 1.778187 | 1.727758 | 1.832112 | 1.333487 | 0.5623704 | 0.5458183 | 0.5787848 |
| HeartDisease | 1.302679 | 1.271646 | 1.337258 | 1.141350 | 0.7676486 | 0.7477991 | 0.7863821 |
| Depression | 1.103801 | 1.081876 | 1.131598 | 1.050620 | 0.9059601 | 0.8837060 | 0.9243204 |
| Asthma | 1.018950 | 1.006201 | 1.057908 | 1.009430 | 0.9814027 | 0.9452615 | 0.9938372 |
| Indi_frequency | 3.020930 | 2.920672 | 3.126420 | 1.738082 | 0.3310239 | 0.3198546 | 0.3423870 |
| Drug_frequency | 1.677728 | 1.631351 | 1.727512 | 1.295271 | 0.5960442 | 0.5788673 | 0.6129889 |

VIF = Variance Inflation Factor; CI = Confidence Interval; SE = Standard Error. VIF > 5 or Tolerance < 0.2 may indicate multicollinearity concerns. In this dataset, all variables show acceptable VIF and Tolerance values, suggesting no severe multicollinearity issues.
